# Clinical and histological features of primary testicular diffuse large B-cell lymphoma: a single center experience in China

**DOI:** 10.18632/oncotarget.19736

**Published:** 2017-07-31

**Authors:** De Zhou, Changqian Bao, Xiujin Ye, Lixia Zhu, Jingjing Zhu, Li Li, Mingyu Zhu, Xiudi Yang, Yanlong Zheng, Xianbo Huang, Mixue Xie, Wanzhuo Xie

**Affiliations:** ^1^ Department of Hematology, the First Affiliated Hospital of Medical School of Zhejiang University, Hangzhou, Zhejiang Province, China; ^2^ Program in Clinical Medicine, School of Medicine, Zhejiang University, Hangzhou, Zhejiang Province, China

**Keywords:** primary testicular lymphoma, diffuse large B-cell lymphoma, RCHOP, intrathecal prophylaxis, radiation therapy

## Abstract

Primary testicular lymphoma (PTL) is a rare and aggressive form of extranodal lymphoma. Approximately 80–98% of PTLs are diffuse large B-cell lymphoma (PT-DLBCL). The prognosis of DLBCL patients has improved with the addition of rituximab to systemic chemotherapy, but outcomes of PT-DLBCL remain poor. This may be explained by the high rate of relapse in the central nervous system (CNS) and contralateral testis. We analyzed 1,132 newly diagnosed DLBCL patients (37 with PT-DLBCL) who were treated at our hospital between January 2009 and December 2014. Twenty-five patients finished follow-up. We analyzed clinical characteristics, response to chemotherapy, overall survival, and relapse in the CNS and contralateral testis. All patients underwent orchiectomy. The median age was 60 (range: 43–82) years. Eleven patients had stage III/IV disease. Five patients experienced CNS relapse, and three experienced relapse in the contralateral testis. Median overall survival (OS) was not reached at the time of reporting. The 3-year OS rate was 57%. None of the nine patients who received radiotherapy to the contralateral testis experienced relapse in that location. Intrathecal prophylaxis did not reduce the risk of CNS relapse. All five patients who experienced CNS relapse had the germinal center B-cell-like subtype of DLBCL.

## INTRODUCTION

Primary testicular lymphoma (PTL) is a rare and aggressive form of extranodal lymphoma. It accounts for 1–2% of all non-Hodgkin's lymphomas (NHLs) and 4% of extranodal lymphomas [1]. Approximately 80–98% of PTLs are diffuse large B-cell lymphoma (PT-DLBCL) [1]. The overall prognosis of DLBCL patients has improved with the addition of rituximab to systemic chemotherapy [2–5]. However, the outcomes of PT-DLBCL patients remain poor owing to the high rate of relapse in the central nervous system (CNS) and contralateral testis [1]. Thus, prophylactic intrathecal chemotherapy and radiation therapy (RT) to the contralateral testis are recommended for these patients [6]. Few studies of PT-DLBCL have been reported [7–11], particularly in China. We retrospectively reviewed the clinical data of DLBCL patients to analyze the clinical features of PT-DLBCL in a Chinese population.

## RESULTS

### Patient characteristics

The clinical characteristics of the patients are summarized in Table 1. The median age was 60 (43–82) years. Bilateral testicular involvement was observed in two patients (8%). Eleven patients (44%) presented with stage III/IV disease. Patient 15 had spleen involvement. No other patients had involvement of extranodal sites. There were six patients (24%) who were diagnosed with the germinal center B-cell-like (GCB) and 19 (76%) with the non-GCB subtype. The mean Ki67 index was 63% (range, 30%–95%). Five patients were CD10-positive and 24 were MUM-1-positive. BCL-2 expression was observed in 18 of 25 patients.

**Table 1:** Baseline characteristics of the patients

Factors	Number	%
Location		
Left	10	40
Right	13	56
Bilateral	2	8
Ann Arbor Stage		
I	11	44
II	3	12
III	9	36
IV	2	8
ECOG PS		
0	12	48
1	11	44
2	2	8
IPI		
0–1	11	44
2	9	36
3	3	12
4–5	2	8
B symptoms		
Present	3	12
Elevated serum LDH	7	28
Elevated β2-microglobulin	4	16
Subtype		
GCB	6	24
Non-GCB	19	76

### Evaluation of treatment and prognosis

**Figure 1:**
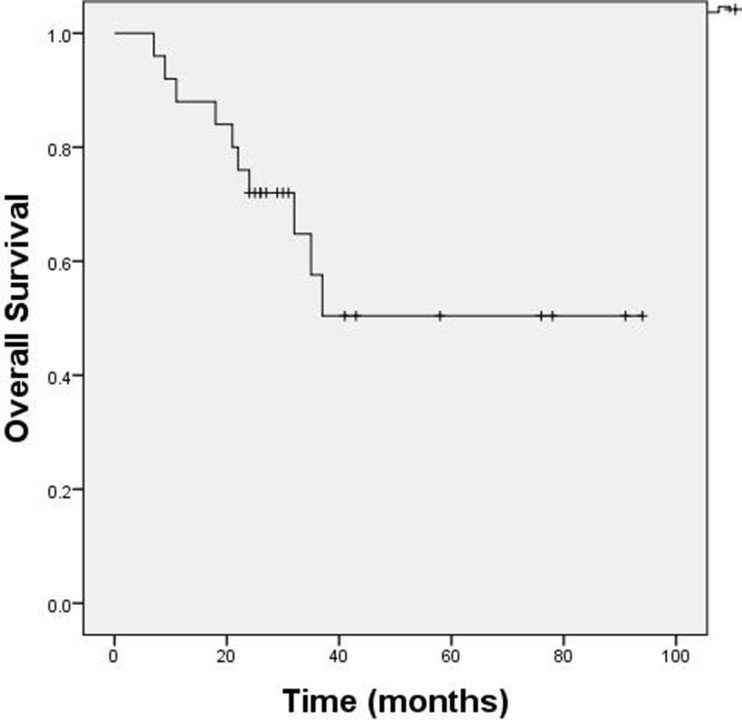
OS of PT-DLBCL patients Median OS was not reached at the time of reporting. The 3-year OS rate was 57%.

Treatment and prognosis data are shown in Table 2. All patients were treated with systemic chemotherapy. Eleven (44%) received CHOP (cyclophosphamide 750 mg/m^2^ I.V., doxorubicin 50 mg/m^2^ intravenously [I.V.], and vindesine 4.0 mg I.V. on day 1, and prednisone, 100 mg orally, on days 1–5 of each cycle), 13 (52%) received RCHOP (rituximab 375 mg/m^2^ I.V. on day 1, cyclophosphamide 750 mg/m^2^ I.V., doxorubicin 50 mg/m^2^ I.V., and vindesine 4.0 mg I.V. on day 2, and prednisone, 100 mg orally, on days 2–6 of each cycle), and one received Hyper-CVAD. Patients received 4–10 courses of chemotherapy and were evaluated after 4 courses. Fifteen patients (60%) achieved CR, eight (32%) achieved PR, and two (8%) had PD. Ten patients (40%) received RT to the contralateral testis (dose, 20–25 Gy). Eighteen patients (72%) were managed with prophylactic CNS intrathecal chemotherapy (50 mg cytarabine, 10 mg methotrexate, and 5 mg dexamethasone). Five patients experienced CNS relapse and three presented with relapse in the contralateral testis. Median OS was not reached at the time of reporting. The 3-year OS rate was 57% (Figure 1).

**Table 2:** Treatment and prognosis of PT-DLBCL patients

Patient no.	Stage	IPI	Regimen	Courses	Response	Prophylactic IT	CNS relapse	Contralateral testis RT	Contralateral testis relapse	PFS (months)	OS (months)
1	I	2	CHOP	6	CR	N	N	Y	N	27	27
2	IV	4	CHOP	4	PR	N	N	N	N	-	21^#^
3	III	2	Hyper-CVAD	4	CR	Y	N	N	N	41	41
4	II	1	RCHOP	8	PR	Y	N	N	N	-	35^#^
5	III	2	RCHOP	8	CR	N	N	N	N	94	94
6	III	2	RCHOP	6	PR	N	N	N	Y	-	78
7	I	0	CHOP	6	CR	Y	Y	N	N	28	32^#^
8	III	2	CHOP	6	PR	Y	N	Y	N	-	24^#^
9	III	2	RCHOP	7	PR	Y	Y	N	N	-	22^#^
10	I	2	CHOP	6	CR	Y	N	Y	N	26	26
11	I	1	RCHOP	5	CR	Y	N	Y	N	31	31
12	I	1	CHOP	4	CR	Y	N	Y	N	29	29
13	IV	3	CHOP	6	PR	Y	Y	N	Y	-	11^#^
14	III	3	CHOP	4	PR	Y	N	Y	N	-	18^#^
15	III	4	RCHOP	4	PD	N	N	N	^*^	-	9^#^
16	I	2	RCHOP	6	CR	Y	N	N	N	30	30
17	II	1	RCHOP	6	CR	Y	N	N	^*^	24	24
18	I	0	RCHOP	6	CR	Y	N	N	N	58	58
19	I	1	CHOP	10	CR	Y	N	N	Y	80	91
20	III	2	CHOP	6	CR	Y	N	N	N	43	43
21	I	0	RCHOP	5	CR	N	N	Y	N	25	25
22	I	0	CHOP	5	PR	Y	N	Y	N	-	26
23	III	3	RCHOP	4	PD	Y	Y	N	N	-	7^#^
24	II	0	RCHOP	7	CR	Y	N	Y	N	13	76
25	I	1	RCHOP	8	CR	N	Y	N	N	34	37^#^

^*^Bilateral testicular infiltration at diagnosis; -Patients who did not achieve CR; # Patients who died (10).

### CNS relapse

There were 18 patients (72%) who received prophylactic intrathecal chemotherapy and four (22.2%) experienced CNS relapse. Only one of the other seven patients (14.3%) experienced CNS relapse. All five patients who experienced CNS relapse had the GCB subtype. We performed immunohistochemical analysis and analyzed cytogenetic alterations in these patients (Table 3). Four patients expressed BCL-2, and two expressed both BCL-2 and MYC. Patients 23 and 25 had *BCL-2* translocations. No patients had *MYC* or *BCL-6* translocations.

**Table 3:** Immunohistochemical analysis and cytogenetic alterations in patients who experienced CNS relapse

Patient No.	Ki-67	BCL-2 Expression	MYC Expression	*BCL-2* Translocation	*MYC* Translocation	*BCL-6* Translocation
7	90%	+	−	−	−	−
9	60%	−	−	−	−	−
13	65%	+	+	−	−	−
23	75%	+	+	+	−	−
25	70%	+	−	+	−	−

### Relapse in the contralateral testis

All nine patients who received RT to the contralateral testis did not experience relapse at this location. Three of the 14 patients who were not treated with RT to the contralateral testis experienced relapse at this site. Two patients were diagnosed with bilateral testicular infiltration.

## DISCUSSION

We analyzed 37 patients with PT-DLBCL who underwent orchiectomy in our Urology department. Twelve patients refused transfer to the Hematology or Chemotherapy departments for additional treatment because of advanced age or financial problems. The other 25 patients with PT-DLBCL were treated with CHOP, RCHOP, or Hyper-CVAD following orchiectomy. All patients completed the median follow-up period of 29 months (range, 7–94 months). Eleven patients (44%) were diagnosed with stage III/IV disease, which was a higher proportion than in other studies [7, 8]. Median OS was not reached at the time of reporting. The 3-year OS rate was 57%.

There were 11 patients who received CHOP and six (54.5%) achieved CR. Thirteen patients received RCHOP and eight (61.5%) achieved CR. The other patient who received hyper-CVAD treatment containing high-dose methotrexate achieved CR. Several retrospective studies [7, 8, 12] have analyzed the effects of rituximab on patient outcomes in PTL and have achieved inconsistent results. The addition of rituximab to systemic chemotherapy improved 5-year OS but not PFS in a study from MD Anderson Cancer Center [7], and PFS in a Chinese study from China [8]. No improvement in patient outcomes was observed in a study from the British Columbia Cancer Agency [12]. Deng et al. [13] demonstrated that rituximab reduced the risk of relapse (*P* = 0.022) and improved both PFS (*P* = 0.012) and OS (*P* = 0.027) in PT-DLBCL patients (5-year PFS, 56% vs. 36%; 5-year OS, 68% vs. 48%). These differences may be explained by the small sample size, biased patient selection, and the lack of data regarding systemic therapy.

The incidence of CNS relapse in an unselected population of DLBCL patients is approximately 5% [14]. In our study, 5 patients experienced CNS relapse (20%), which is consistent with data from previous studies (9%–35%) [7, 15]. Patients who experience CNS relapse have a poor prognosis. Therefore, we evaluated the effects of prophylactic CNS intrathecal chemotherapy. There were 18 patients (72%) who received prophylactic CNS intrathecal chemotherapy and four (22.2%) experienced CNS relapse. Only one of the other seven patients experienced CNS relapse (14.3%). Intrathecal prophylaxis did not reduce the risk of CNS relapse. The rate of CNS relapse was not affected by the administration of intrathecal prophylaxis, consistent with retrospective studies of PTL conducted at the IELSG and MD Anderson Cancer Center [16, 17]. Interestingly, all five patients who experienced CNS relapse had the GCB subtype. The non-GCB subtype was previously associated with a high risk of CNS relapse in DLBCL patients. Cell of origin, serum LDH level, extranodal involvement sites, Ann Arbor stage, IPI score, and Ki67 index were also associated with the rate of CNS relapse. Kerry et al. [18] reported that dual expression of MYC and BCL-2 and *BCL-2*, *MYC*, and *BCL-6* translocations were associated with a high risk of CNS relapse in DLBCL. There were no differences in LDH level, extranodal involvement sites, IPI score, or Ki67 index among these five patients. We observed BCL-2 expression in four patients, and both BCL-2 and MYC expression in two patients. Two patients had *BCL-2* translocations, while no patients had *MYC* or *BCL-6* translocations. This could be explained by CNS relapse.

All nine patients who received RT to the contralateral testis did not experience relapse at this location. Three of the 14 patients who did not receive RT to the contralateral testis experienced relapse at this site. Two patients were diagnosed with bilateral testicular infiltration. A potential sanctuary site formed by the blood-testis barrier renders testicular tumors inaccessible to systemic chemotherapy, but can be removed by orchiectomy. However, because most patients experience relapse within two years of treatment, orchiectomy alone is not a definitive treatment [19]. The IELSG study reported that the continuous risk of relapse in the contralateral testis was 15% at 3 years and 42% at 15 years in patients who were not treated with prophylactic testicular irradiation. Prophylactic testicular irradiation was shown to reduce the rate of testicular relapse from 35% to 8% [17].

Our retrospective study has several limitations. The small study population, variable treatment protocols, and relatively short follow-up period made it difficult to identify relevant prognostic factors. Additionally, OS was worse than in other retrospective studies because many patients had advanced stage disease and high IPI scores. This could be explained by a lack of financial support, advanced age, and shame associated with visiting a doctor for a genital-related disease.

According to the 2016 version of the World Health Organization classification of lymphoid malignancies, PT-DLBCL is classified under DLBCL not otherwise specified. However, our findings suggest that PT-DLBCL is a unique subtype of DLBCL. Although most patients (60% in our study and 86–97% in other trials [9, 13, 19]) achieved CR after systemic chemotherapy, PT-DLBCL was associated with a high risk of relapse. Thus, additional clinical trials are required to improve the treatment of these patients.

## MATERIALS AND METHODS

### Patients

We reviewed the clinical data of 1,132 newly diagnosed patients with DLBCL who were treated at the First Affiliated Hospital of Medical School of Zhejiang University between January 2009 and December 2014. Of these patients, 37 had primary involvement of the testis. All the patients underwent orchiectomy and a comprehensive examination including blood count, liver and kidney function, lactate dehydrogenase (LDH), β2-microglobulin, and coagulation function tests. All diagnoses were confirmed by histology according to the World Health Organization Classification. Twelve patients did not receive either chemotherapy or RT. Complete clinical profiles were obtained for the remaining 25 patients who completed follow-up. Clinical staging and diagnostic methods included a clinical history and physical examination, positron emission tomography-computed tomography, chest, abdominal, and pelvic computed tomography, color Doppler ultrasound, and bone marrow aspiration and biopsy. Tumor response was classified as complete response (CR), unconfirmed complete response (CRU), partial response (PR), stable disease (SD), or progressive disease (PD) according to the International Workshop Criteria [20].

### Statistical analysis

Statistical analysis was performed on follow-up data from January 2009 to October 2016 using SPSS19.0. Overall survival (OS) was defined as the time from diagnosis to death. Progression-free survival (PFS) was defined as the time from diagnosis to either disease progression or death. OS was assessed using the Kaplan-Meier method.

## References

[1] Cheah CY, Wirth A, Seymour JF (2014). Primary testicular lymphoma. Blood.

[2] Coiffier B, Lepage E, Briere J, Herbrecht R, Tilly H, Bouabdallah R, Morel P, Van Den Neste E, Salles G, Gaulard P, Reyes F, Lederlin P, Gisselbrecht C (2002). CHOP chemotherapy plus rituximab compared with CHOP alone in elderly patients with diffuse large-B-cell lymphoma. N Engl J Med.

[3] Pfreundschuh M, Trümper L, Osterborg A, Pettengell R, Trneny M, Imrie K, Ma D, Gill D, Walewski J, Zinzani PL, Stahel R, Kvaloy S, Shpilberg O (2006). CHOP-like chemotherapy plus rituximab versus CHOP-like chemotherapy alone in young patients with good-prognosis diffuse large-B-cell lymphoma: a randomised controlled trial by the MabThera International Trial (MInT). Group. Lancet Oncol.

[4] Pfreundschuh M, Schubert J, Ziepert M, Schmits R, Mohren M, Lengfelder E, Reiser M, Nickenig C, Clemens M, Peter N, Bokemeyer C, Eimermacher H, Ho A (2008). Six versus eight cycles of bi-weekly CHOP-14 with or without rituximab in elderly patients with aggressive CD20+ B-cell lymphomas: a randomised controlled trial (RICOVER-60). Lancet Oncol.

[5] George A, Tam CS, Seymour JF (2013). High-risk diffuse large B-cell lymphoma: can we do better than rituximab, cyclophosphamide, doxorubicin, vincristine and prednisone?. Leuk Lymphoma.

[6] Visco C, Medeiros LJ, Mesina OM, Rodriguez MA, Hagemeister FB, McLaughlin P, Romaguera JE, Cabanillas F, Sarris AH (2001). Non-Hodgkin's lymphoma affecting the testis: is it curable with doxorubicinbased therapy?. Clin Lymphoma.

[7] Mazloom A, Fowler N, Medeiros LJ, Iyengar P, Horace P, Dabaja BS (2010). Outcome of patients with diffuse large B-cell lymphoma of the testis by era of treatment: the M. D. Anderson Cancer Center experience. Leuk Lymphoma.

[8] Wang Y, Li ZM, Huang JJ, Xia Y, Li H, Li YJ, Zhu YJ, Zhao W, Xia XY, Wei WX, Huang HQ, Lin TY, Jiang WQ (2013). Three prognostic factors influence clinical outcomes of primary testicular lymphoma. Tumour Biol.

[9] Kim J, Yoon DH, Park I, Kim S, Park JS, Lee SW, Huh J, Park CS, Suh C (2014). Treatment of primary testicular diffuse large B cell lymphoma without prophylactic intrathecal chemotherapy: a single center experience. Blood Res.

[10] Lokesh KN, Sathyanarayanan V, Kuntegowdanahalli CL, Suresh TM, Dasappa L, Kanakasetty GB (2014). Primary Diffuse large B-Cell lymphoma of testis: A single centre experience and review of literature. Urol Ann.

[11] Vitolo U, Chiappella A, Ferreri AJ, Martelli M, Baldi I, Balzarotti M, Bottelli C, Conconi A, Gomez H, Lopez-Guillermo A, Martinelli G, Merli F, Novero D (2011). First-line treatment for primary testicular diffuse large B-cell lymphoma with rituximab-CHOP, CNS prophylaxis, and contralateral testis irradiation: final results of an international phase II trial. J Clin Oncol.

[12] Kridel R, Telio D, Villa D, Sehn LH, Gerrie AS, Shenkier T, Klasa R, Slack GW, Tan K, Gascoyne RD, Connors JM, Savage KJ (2017). Diffuse large B-cell lymphoma with testicular involvement: outcome and risk of CNS relapse in the rituximab era. Br J Haematol.

[13] Deng L, Xu-Monette ZY, Loghavi S, Manyam GC, Xia Y, Visco C, Huh J, Zhang L, Zhai Q, Wang Y, Qiu L, Dybkær K, Chiu A (2016). Primary testicular diffuse large B-cell lymphoma displays distinct clinical and biological features for treatment failure in rituximab era: a report from the International PTL Consortium. Leukemia.

[14] Zhang J, Chen B, Xu X (2014). Impact of rituximab on incidence of and risk factors for central nervous system relapse in patients with diffuse large B-cell lymphoma: a systematic review and meta-analysis. Leuk Lymphoma.

[15] Kridel R, Dietrich PY (2011). Prevention of CNS relapse in diffuse large B-cell lymphoma. Lancet Oncol.

[16] Seymour JF, Solomon B, Wolf MM, Janusczewicz EH, Wirth A, Prince HM (2001). Primary large-cell non-Hodgkin's lymphoma of the testis: a retrospective analysis of patterns of failure and prognostic factors. Clin Lymphoma.

[17] Zucca E, Conconi A, Mughal TI, Sarris AH, Seymour JF, Vitolo U, Klasa R, Ozsahin M, Mead GM, Gianni MA, Cortelazzo S, Ferreri AJ, Ambrosetti A (2003). Patterns of outcome and prognostic factors in primary large-cell lymphoma of the testis in a survey by the International Extranodal Lymphoma Study Group. J Clin Oncol.

[18] Savage KJ, Slack GW, Mottok A, Sehn LH, Villa D, Kansara R, Kridel R, Steidl C, Ennishi D, Tan KL, Ben-Neriah S, Johnson NA, Connors JM (2016). Impact of dual expression of MYC and BCL2 by immunohistochemistry on the risk of CNS relapse in DLBCL. Blood.

[19] Vitolo U, Ferreri AJ, Zucca E (2008). Primary testicular lymphoma. Crit Rev Oncol Hematol.

[20] Cheson BD, Pfstner B, Juweid ME, Gascoyne RD, Specht L, Horning SJ, Coiffier B, Fisher RI, Hagenbeek A, Zucca E, Rosen ST, Stroobants S, Lister TA (2007). Revised response criteria for malignant lymphoma. J Clin Oncol.

